# Magnetism in the p-type Monolayer II-VI semiconductors SrS and SrSe

**DOI:** 10.1038/srep45869

**Published:** 2017-04-05

**Authors:** Heng-Fu Lin, Woon-Ming Lau, Jijun Zhao

**Affiliations:** 1 Beijing Computational Science Research Center, Beijing, 100094, China

## Abstract

Using density functional theory calculations, we study the electronic and magnetic properties of the p-type monolayer II-VI semiconductors SrX (X = S,Se). The pristine SrS and SrSe monolayers are large band gap semiconductor with a very flat band in the top valence band. Upon injecting hole uniformly, ferromagnetism emerges in those system in a large range of hole density. By varying hole density, the systems also show complicated phases transition among nonmagnetic semiconductor, half metal, magnetic semiconductor, and nonmagnetic metal. Furthermore, after introducing p-type dopants in SrS and SrSe via substitutionary inserting P (or As) dopants at the S (or Se) sites, local magnetic moments are formed around the substitutional sites. The local magnetic moments are stable with the ferromagnetic order with appreciable Curie temperature. The ferromagnetism originates from the instability of the electronic states in SrS and SrSe with the large density of states at the valence band edge, which demonstrates a useful strategy for realizing the ferromagnetism in the two dimensional semiconductors.

Since the successful realization of graphene in experiments^1^,^2^,^3^, two dimensional (2D) materials have attracted a lot of attentions. Their extraordinary properties make them promising materials not only for exploring novel physical phenomena^4^,^5^,^6^,^7^,^8^,^9^,^10^,^11^,^12^ but also as building blocks of device applications^13^,^14^,^15^. Usually, 2D materials can be easily integrated into semiconductor devices^16^. In the emerging field of spintronics, the marriage between the world of 2D semiconductors and the world of magnetism is very important. Despite the great success of 2D materials, magnetism in 2D semiconductors remains largely unexplored. To realize the magnetic semiconductor, the most popular way is to incorporate the magnetic transition-metal atoms^17^,^18^,^19^. Alternatively, recent theoretical and experimental works revealed that the ferromagnetism can be also realized in some *d*^0^ systems^20^,^21^,^22^,^23^ without the help of transition metal atoms with partially filled *d* or *f* shell. This type of “*d*^0^ ferromagnetism” provides a new opportunity for searching high-temperature spintronic materials in 2D materials since that the *p* bands can be spontaneously polarized, giving a ferromagnetic state that can be tuned easily. For instance, by introducing nonmagnetic impurities, creating vacancies, or injecting uniform holes, magnetism have been induced in the monolayer *h*-BN^24^, graphene^25^, silicene^26^, MoS_2_^27^ and GaSe^28^.

Recently, binary 2D semiconductors such as III-V compounds (BN), IV-VI compounds (MoS_2_) are promising playgrounds for designing future devices. Considering the spin and charge degrees of freedom may couple with each other, these materials could show many interesting properties, such as large band gap^24^, exotic spin and pseudospin physics^27^. The SrX (X = S,Se) are the group II-VI binary systems with planar honeycomb structure similar to *h*-BN, while the sixfold symmetry of the honeycomb lattice is broken due to the existence of the A/B sublattices^29^. Those 2D II-VI structures could be fabricated on a substrate, just like the silicene, germanene, and *h*-BN^30^,^31^. Like the traditional II-VI three-dimensional (3D) compounds with suitable band gaps which are important in electronics and optoelectronics^32^,^33^, the SrS and SrSe monolayer sheets are thermally and dynamically stable^29^ and also possess a gap near visible light. Besides, the 2D SrX (X = S, Se) possesses a flat valence band, which gives rise to a large density of states at the valence band edge and make them an excellent candidate for realizing Stoner ferromagnetism. Thus, realizing the dilute magnetic semiconductors based on SrX (X = S,Se) are likely to be easier than other 2D semiconductors with possible doping and chemical modifications.

In this paper, we carry out first-principles calculations to explore the magnetism and its origin in the *p*-type monolayer sheets of SrS and SrSe. The pristine SrS and SrSe monolayers are semiconductor with a flat band in the top valence band, which gives rise to a large density of states near the Fermi level. Both uniformly hole doping and *p*-type substitutional dopant can induce ferromagnetism in the SrS and SrSe monolayers, paving a way for experimental verification and applications in spintronic devices.

## Method

Our calculations are performed within density functional theory (DFT) using the projector augmented wave (PAW) potentials^34^,^35^ and a plane-wave basis set, as implemented in Vienna ab initio simulation package (VASP)^36^,^37^. Exchange-correlation potentials are treated within the generalized gradient approximation (GGA) of Perdew, Burke, and Ernzerhof (PBE) parameterization^38^. The possible underestimations of band gap and magnetic moment within GGA is checked by a nonlocal Heyd-Scuseria-Ernzerhof functional (HSE06)^39^. For the present calculations, Sr 4s^2^4p^6^5s^2^, S 3s^2^3p^4^, and Se 4s^2^4p^4^ electrons are considered as the valence electrons. A vacuum space of 15 Å between layers is used to avoid the interactions between the layer and its periodic images. We use a cutoff energy of 500 eV for plane-wave basis set and sample the Brillouin zone using a 12 × 12 × 1 k-point grid generated with Monkhorst-Pack scheme^40^ for the primitive cell. Holes are injected by removing electrons from the system and using a homogeneous background charge to maintain charge neutrality. For various doping conditions, a 18 × 18 × 1 k-point grid is adopted. For the substitutional doping, we adopt the 4 × 4 × 1 supercell, which is large enough to avoid the interaction between the dopants. A set of 3 × 3 × 1 k-point is used for the dopant calculation. The 2D crystal structures are fully relaxed until the residual forces on each atom are less than 1.0 × 10^−2^ eV/Å and the total energy less than 1.0 × 10^−4^ eV.

**Figure 1:**
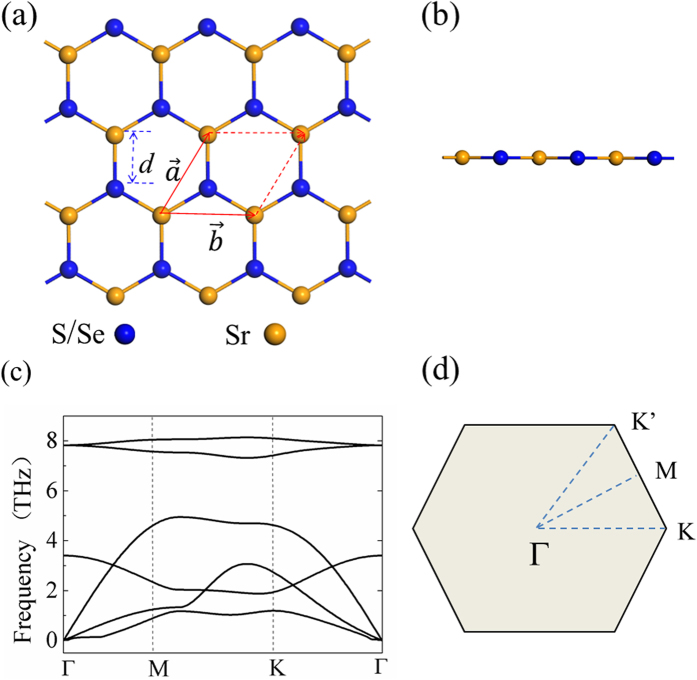
(**a**) Top view of the honeycomb structure for 2D II-VI semiconductors SrX (X = S,Se), where 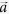 and 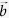
are lattice vectors, *d* is the shortest bond length between two types of atoms. (**b**) Side view of SrX, where the group II and the group VI atomic are located in the same plane without bucking, just like graphene. (**c**) Phonon dispersion curves of SrS monolayer. The absence of imaginary frequency demonstrates its dynamic stability. (**d**) Brillouin zone (BZ) of 2D II-VI semiconductors. *Γ, M, K*, and *K*’ denote the high-symmetry points in the BZ.

The crystal structure of SrX (X = S, Se) is shown in Fig. 1, which has a planar hexagonal structure made of group IV and VI elements, similar to graphene and *h*-BN. The primitive unit cell of SrX contains one group II atom (i.e., Sr) and one group VI atom. The SrX monolayer has the D_3h_ point-group symmetry. The structure consists of the A and B sublattices. For example, we can assume that the Sr atoms reside at the A sites, and the group VI (S, Se) atoms reside at the B sites, respectively. The inversion symmetry and sixfold symmetry of the honeycomb lattice is broken due to the asymmetry of the A/B sublattices. The optimized structural parameters are: lattice constant a = 4.85 Å and bond length d = 2.80 Å for SrS; lattice constant a = 5.05 Å and bond length d = 2.93 Å for SrSe. The dynamic stability of SrS and SrSe has been investigated by calculating its phonon spectrum. Imaginary frequencies are not found in the phonon dispersion, which indicates these structures are stable. The calculated phonon dispersion of SrS monolayer is shown in Fig. 1(c).

## Results

### Electronic band structures

**Figure 2:**
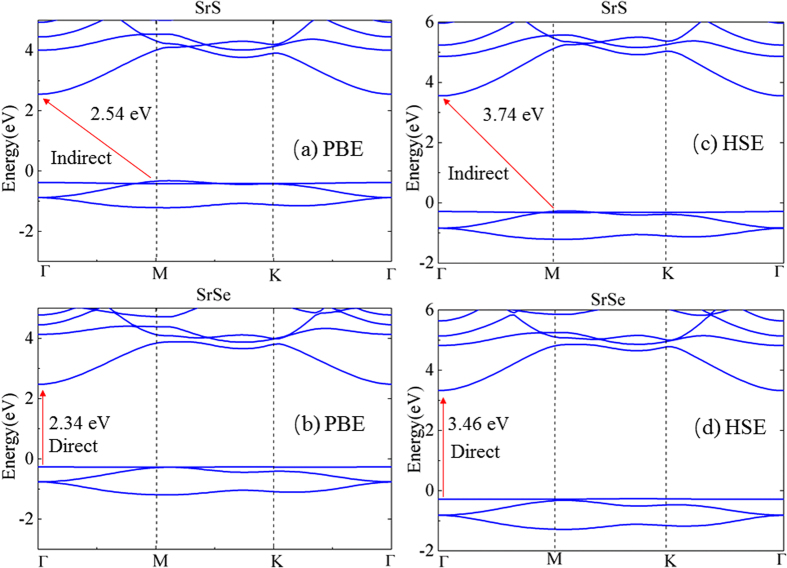
Calculated band structures of pristine monolayers (**a**) SrS and (**b**) SrSe using the PBE functional. The results for (**c**) SrS and (d) SrSe using the HSE functional are also given. The zero energy is set at the VBM.

The band structures of SrS and SrSe monolayers from PBE calculations are shown in Fig. 2(a) and (b), respectively. SrS monolayer sheet is an indirect-band-gap semiconductor, in which the conduction band minimum (CBM) locates at the Γ point of the BZ center and the valence band maximum (VBM) locates at the M point. However, SrSe monolayer sheet is a direct-band-gap semiconductor at the Γ point. The 2D II-VI semiconductors have a noticeably large band gap and large iconicity, as compared to its group IV counterparts like silicene and germanene. The PBE band gaps for the SrS and SrSe systems are 2.54 eV and 2.34 eV, respectively. Specifically, our calculations show that there is flat band with little k dependence in the top valence band in both monolayer systems. To further validate these results, we have carried out band structure calculations using the HSE06 functional (see Fig. 2(c) and (d)), which is known to improve the description of band gap of a semiconductor. The HSE06 band gaps for the SrS and SrSe systems are 3.74 eV and 3.46 eV, respectively. Although the band gap is enlarged by HSE06, the band dispersion remains unchanged with the flat band in the valence band edge. This band dispersion behavior is distinctly different from the 2D semiconductor MoS_2_ with direct band gap and 2D insulator h-BN with indirect band gap. Such a flat band could give rise to a sharp van Hove singularity in the density of states (DOS). The large DOS near the valence band edge would lead to instabilities to different phases, such as ferromagnetism, superconductivity, and charge density wave^41^,^42^,^43^. Moreover, the essential feature of valence band, such as the flat band and large band gap, can be directly detected using the angle-resolved photoemission spectroscopy (ARPES)^44^,^45^,^46^.

**Figure 3:**
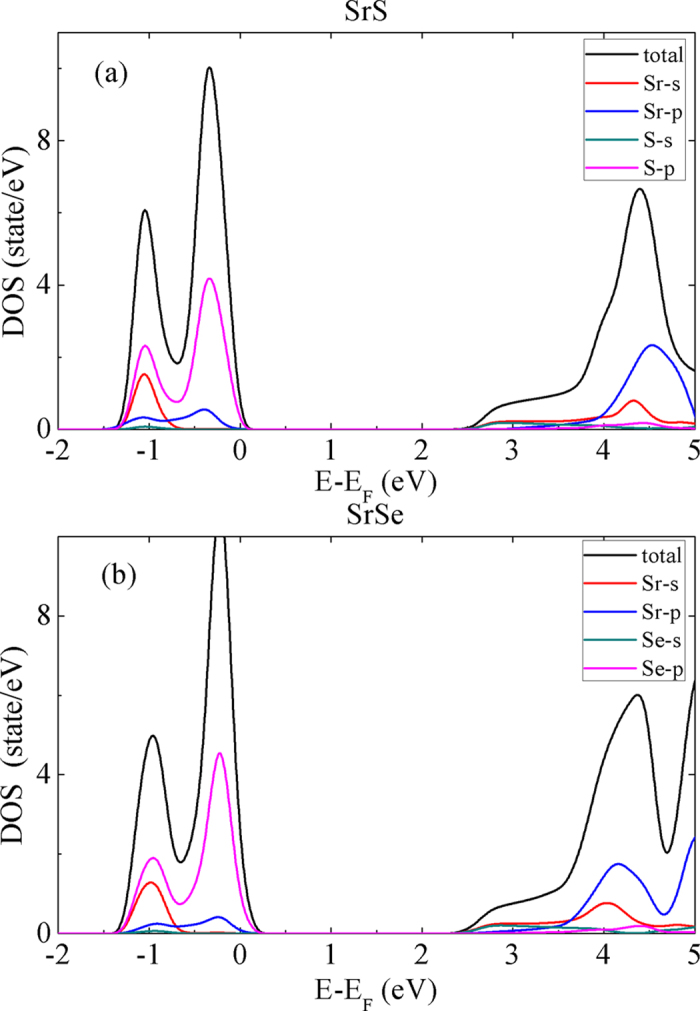
Total and partial density of states of pristine monolayers of (**a**) SrS and (**b**) SrSe using the PBE functional. The zero energy is set at the VBM.

The total and partial electron density of states are given in Fig. 3. Obviously, there is a sharp peak in the density of states almost exactly at the valence band edge. From the partial DOS, we can see that the state near the VBM is mainly composed of Se p or S p orbitals, with a small contribution from Sr p orbitals. On the other hand, CBM is composed of Sr p and Sr s orbitals mixed with small portion of S s or Se s orbitals.

### Hole-induced ferromagnetism

**Figure 4:**
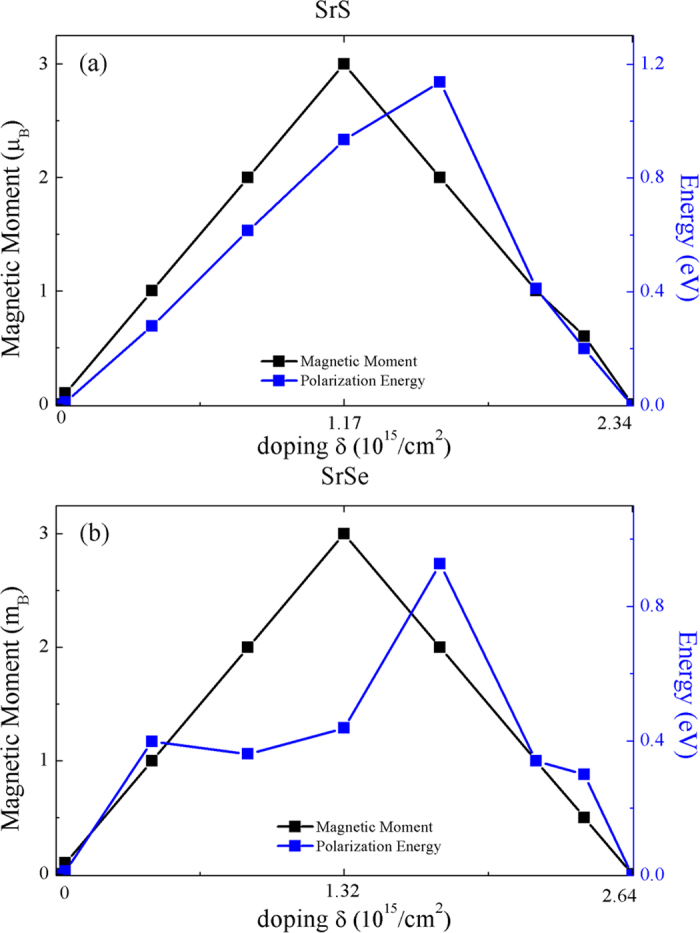
The magnetic moment, spin-polarization energy in the out-of-plane spin-polarized ferromagnetic state as a function of the carrier density for the hole-doped monolayers of (**a**) SrS and (**b**) SrSe. The threshold hole density for ferromagnetism in both systems is about 4.0 × 10^12^/cm^2^.

Although pristine SrS and SrSe monolayers are nonmagnetic, our spin-polarized calculations show that those systems spontaneously develop a ferromagnetic ground state with some amount of hole doping. Figure 4 shows the calculated spin moment per cell as a function of the hole density δ. In both systems, for very small hole density, the magnetic moment is zero, the systems continue to be nonmagnetic. When the hole density is large enough, the system become spin polarized. The threshold hole density for ferromagnetism in both systems is about 4.0 × 10^12^/cm^2^. At the spin polarized region, with the increasing of the hole density δ, the magnetic moment increases monotonically until δ reaches a critical value δ_c1_, then it decreases monotonically back to zero at another critical value δ_c2_. The critical values are δ_c1_ = 1.17 × 10^15^/cm^2^, δ_c2_ = 2.34 × 10^15^/cm^2^ in SrS, and δ_c1_ = 1.32 × 10^15^/cm^2^, δ_c2_ = 2.64 × 10^15^/cm^2^ in SrSe, respectively.

In order to examine the stability of the spin-polarized state, we also calculate the spin polarization energy *E*_P_. The spin polarization energy *E*_P_ is described as: *E*_P_ = *E*_non_ − *E*_FM_, where *E*_non_ and *E*_FM_ are the total energy of the nonmagnetic and ferromagnetic phases, respectively. The spin-polarized energy per cell as a function of the hole density is also shown in Fig. 4. Like the magnetic moment, the spin-polarization energy is also highly dependent on the hole density. For the SrS, the spin-polarization energy increases monotonically to 0.4 eV/cell at δ = 1.51 × 10^15^/cm^2^ and then decreases monotonically back to zero at δ = 2.34 × 10^15^/cm^2^. For SrSe, the spin-polarization energy increases non-monotonically to 0.33 eV/cell at δ_c1_ = 1.76 × 10^15^/cm^2^, and then decreases monotonically back to 0 at δ = 2.64 × 10^15^/cm^2^. Roughly speaking, the magnetic moment and spin-polarization energy show consistent trend with the hole density changing. With intermediate level of hole doping, the system possesses a high spin-polarization energy, which endows a more stable ferromagnetic state with relatively high Curie temperature.

The physical origin of the ferromagnetic phase over the nomagnetic phase in SrS and SrSe monolayers upon hole doping can be understood from the Stoner criterion^47^,^48^. As known, the susceptibility of a system is given by a formula on the basis of random phase approximation (RPA): χ = χ_0_/[1− *I*·*N(E*_F_)], where χ_0_ is the bare Pauli susceptibility, χ_0_ = μ_B_*N(E*_F_), *N(E*_F_) is the non-spin-polarized density of states at the Fermi level *E*_F_, and *I* is the Stoner exchange integral. *I* has been calculated for most elements in the periodic table and its value is typically around 0.7 ~ 0.8 eV^49^ without much variation. This formula reflects the major effect of electronic band structure, but neglects the effects of spin-fluctuations which can renormalize the spin susceptibility. If the system satisfies the Stoner criteria *I*·*N(E*_F_) > 1, spontaneous ferromagnetism occurs. In the SrX systems, when hole density is zero, the Fermi level *E*_F_ lies just at the VBM and *N(E*_F_) is close to zero, resulting in zero magnetic moment because *I*·*N(E*_F_) < 1. When there are more holes, the Fermi level moves into the valence band and *N(E*_F_) increases, When the hole concentration becomes large enough so that *I*·*N(E*_F_) > 1, the system can lower its total energy by exchange splitting of the bands and become ferromagnetic. In our calculation, holes are artificially injected by removing electrons from the system. Experimentally, the holes can be injected by ion liquid gating or back-gate gating. For instance, tunable doping carrier densities have been achieved in graphene by ion liquid gating^50^,^51^,^52^ and in transition metal dichalcogenide monolayers by back-gate gating, respectively^52^,^53^.

**Figure 5:**
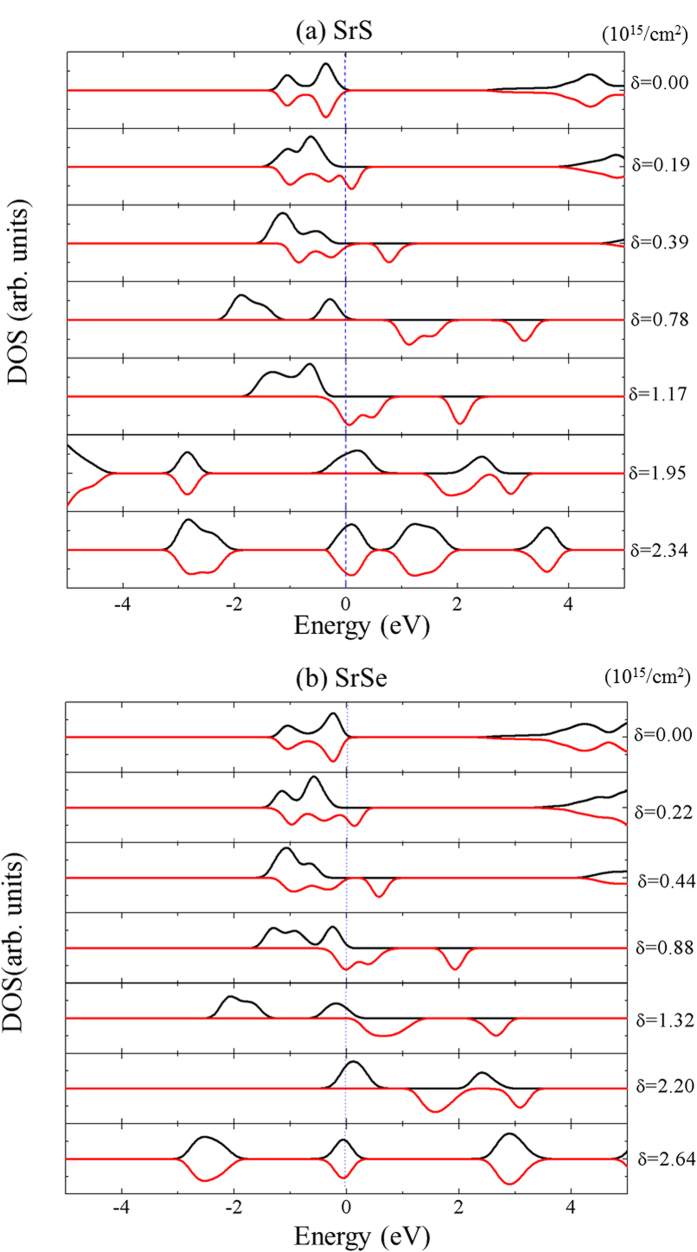
Spin-resolved total DOS of SrS (**a**) and SrSe (**b**) around the Fermi level as a function of the hole density δ. Vertical dotted lines denote the Fermi level. Positive values of DOS correspond to the majority-spin electrons and negative values to the minority-spin electrons.

The hole induced-ferromagnetism will produce an effective Zeeman exchange field. In turn, the exchange field in the ferromagnetic phase will lead to exchange splitting of the bands from the two spin species. The spin-resolved DOS of both SrS and SrSe systems for various hole density δ are depicted in Fig. 5. We find that the electronic and magnetic properties of both systems can be tuned by the hole density δ, creating various interesting phases like nonmagnetic semiconductor, half metal, magnetic semiconductor, and nonmagnetic metal. As a representative example, the phase transitions as a function of hole density δ in the SrSe monolayer system are carefully determined. When 0 < δ < 4.0 × 10^12^/cm^2^, the DOS of the both spin channels are gapped at the Fermi level without spin exchange splitting, the system is a nonmagnetic semiconductor. For 4.0 × 10^12^/cm^2^ < δ < 0.35 × 10^15^/cm^2^ and 0.8 × 10^15^/cm^2^ < δ < 2.54 × 10^15^/cm^2^, the DOS shows a gap in the minority spin channel, however do not have gap in the majority spin channel, indicating that the system becomes a half metal. Then, at 0.35 × 10^15^/cm^2^ < δ < 0.8 × 10^15^/cm^2^, the DOS of both spin channels has a finite gap (in the order of magnitude of a few eV) with exchange splitting, thus the system becomes a magnetic semiconductor. At last, for δ > 2.54 × 10^15^/cm^2^, the DOS of both spin channels are gapless without exchange splitting, the system is a non-magnetic metal.

### Magnetism from p-type substitutional dopants

**Figure 6:**
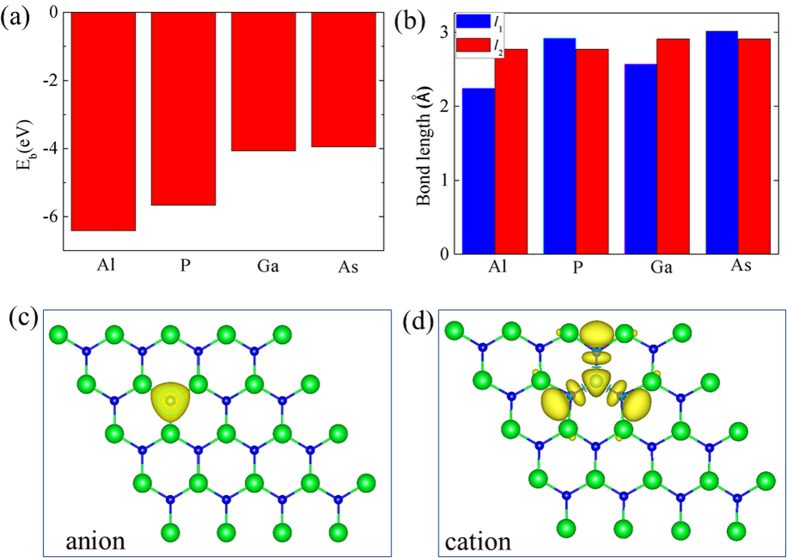
(**a**) The binding energy of the dopant atoms (D) on monolayer GrX. (**b**) The length of D-X bond *l*_1_ and Sr-X bond *l*_2_ of the doped monolayer GrX. (**c**) and (**d**) Spin density (ρ^↑^-ρ^↓^) for an anion substitutional P (or As) and a cation substitutional Al (or Ga) in a 4 × 4 unit cell of SrS (or SrSe) monolayer. Yellow and cyan isosurfaces represent positive and negative spin density (±0.054 e/Ả), respectively.

The above discussions show that it is possible to obtain the stable magnetic moment and ferromagnetism only by injecting holes uniformly into the SrX monolayers. It is known that the incorporation of p-type dopants is also a useful means to induce magnetic states. For generality, we consider the case that the hole doping occurs at both the cation and anion sites. For cation-site doping case, we use a supercell by 4 × 4 unit cells of SrX, with one of the Sr atom being substituted by a Al (or Ga) atom; for the anion-site doping case, one of the Se (or S) atom is substituted by a As (or P) atom. The binding energy *E*_b_ is described as *E*_b_ = *E*_tot_(SrX + D) − (*E*_vac_ + *E*_D_), where *E*_vac_ and *E*_D_ are energies of the defective SrX monolayer with vacancy and isolated substitutional atom D, respectively. The binding energies of the dopant atoms (D) on monolayer SrX are shown in Fig. 6(a). All the binding energies are negative indicating that all the extrinsic atoms are energetically favorable to the vacant sites. The structure relaxation shows that the three D-X bonds have an equivalent length, the doped monolayer SrX do not show a Jahn-Teller distortion. The length of D-X bond (*l*_1_) and Sr-X bond (*l*_2_) of the doped monolayer SrX are shown in Fig. 6(b).

We now turn to the magnetic properties of the doped SrS and SrSe systems. For the cation-site doping case, the resulting total magnetic moment for this system is 1.0 μ_B_, equal to the number of holes. The local magnetic moment is 0.25 μ_B_ at the Ga site and 0.2 μ_B_ on each of the three nearest Se atom sites, 0.025 μ_B_ at each of the six nearest Sr atom sites. The spin density (ρ^↑^-ρ^↓^) around a substitutional Al (or Ga) atom is shown in Fig. 6(d). The hole wave function distributes mostly on the three neighboring trigonal S(or Se) sites. The magnetization through cation-site doping is achievable, but not very efficient because the holes are distributed into several neighboring anion sites. Hence, we focus on the case of the hole doping at the anion sites. Figure 6 Displays the spin density (ρ^↑^-ρ^↓^) around a substitutional P (or As) atom in a 4 × 4 cell of monolayer SrX. When a neutral S (or Se) atom is substituted by a P (or As) atom, one hole is introduced with the hole wave function distributed mostly on the doping site of P (or As). The total magnetic moment within the supercell is 1.0 μ_B_. Population analysis shows that 0.95 μ_B_ is located at the As site, and 0.013 μ_B_ on each of the three nearest neighbor Sr sites. The neighboring Sr atoms are ferromagnetically coupled to the As dopant. Nearly identical results on the total and on-site magnetic moments are found by HSE06 calculations.

**Figure 7:**
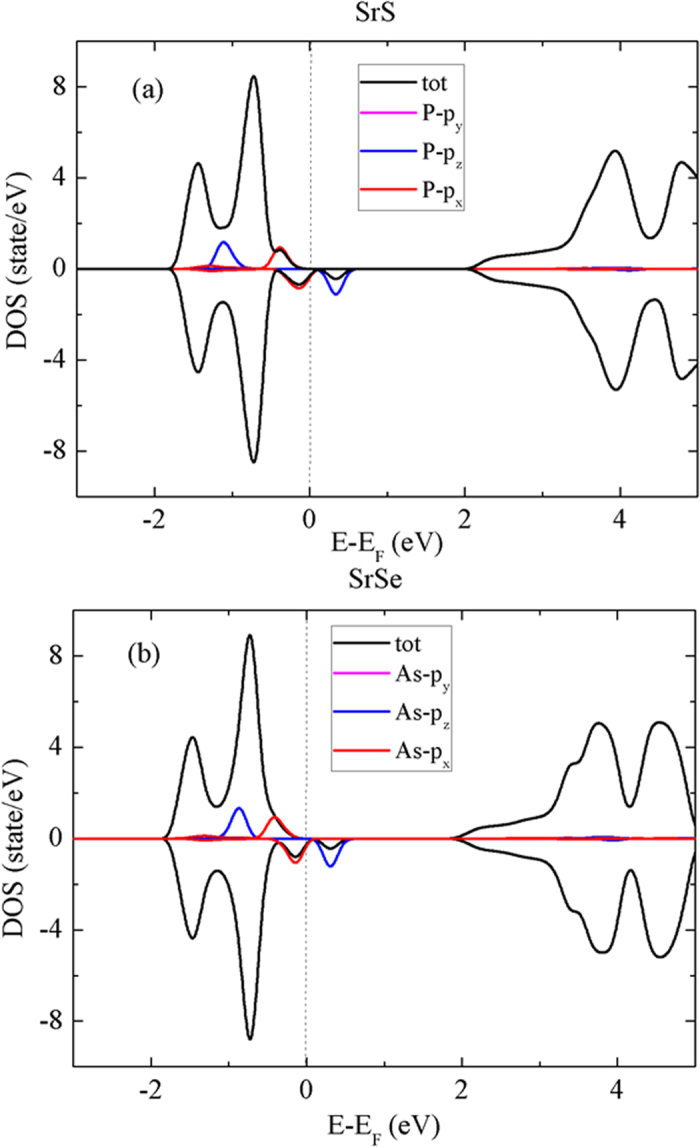
The total density of states (TDOS) and partial density of states (PDOS) for (**a**) P doped monolayer SrS and (**b**) As doped monolayer SrSe calculated using PBE functionals. The mid-gap states emerge in the doped monolayer. The Fermi level of the doped monolayer is set to zero, and the TDOS scale is rescale by sixteen times to compare with the PDOS.

Additional insight into the magnetism and electronic structure of the P (or As) doped SrS (or SrSe) monolayer can be obtained from the electron density of states (DOS) in Fig. 7(a) and (b). The resulting magnetic moments can be understood in light of symmetry consideration. When the As (or P) atom sits at the high-symmetry site of SrX, the p orbital of As (or P) atom splits into two groups under the C_3υ_ symmetric crystal field^54^: the p_z_ state corresponds to *A*_*1*_ group, the twofold degenerate *E* group is related to p_x_ and p_y_. The fully occupied *E* state lies below the Fermi level. Due to spin polarization, the *A*_1_ state splits into (unoccupied) spin-down *A*_1↓_ and (occupied) spin-up *A*_1↑_. Therefore, an As (or P) impurity should result in a net local moment of 1.0 μ_B_.

The possible magnetic state, either ferromagnetic (FM) or anti-ferromagnetic (AFM) is studied by calculating the total energy difference of the two configurations at the same impurity separation, defined as Δ*E*_FM−AFM_. To investigate coupling between these hole-induced local magnetic moments, we employ the 8 × 4 supercell with one defect in each 4 × 4 subsupercell and the 12 × 6 supercell with one defect in each 6 × 6 subsupercell. When the system is a FM insulator, the effective Heisenberg spin model is defined as *H* = −*J*_eff_Σ_<ij>_*S*_i_·*S*_j_, where *J*_eff_ is the effective exchange coupling strength of the nearest-neighbor dopant atoms at site *i* and *j*, and *S*_i_ is the spin of the dopant at lattice site *i*. Considering nearest-neighbor interactions, the energy difference of AFM and FM states is Δ*E*_FM−AFM_ = *E*_FM_ − *E*_AFM_ = 2*J*_eff_*S*^2^. The energy difference Δ*E* and the exchange coupling parameter *J*_eff_ for SrS and SrSe monolayers are shown in Table 1. In both cases, the FM state has a lower energy than the AFM state; hence the system is stable at FM states with positive J_eff_. The important parameter of a dilute magnetic semiconductor (DMS) is the Curie temperature (*T*_C_), blow which the system develops a long-range ferromagnetic ordering. One can estimate T_C_ based on the mean-field approximation (MFA) and Heisenberg model via: 
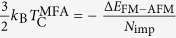
, where *k*_B_ is the Boltzmann constant, and *N*_imp_ = 2 is the number of the P (or As) impurities in the supercell. However, it is well known that the magnetic ordering in DMS is strongly influenced by percolation; the mean-field approximation cannot capture this behavior and tends to systematically overestimate the Currie temperature in these systems^55^,^56^,^57^,^58^. Therefore, to allow for a proper estimation of the Curie temperature, we use an empirical relation *T*_C_/*T*_C_^MFA^ = 0.61^59^. The determined Curie temperatures *T*_C_ and *T*_C_^MFA^ are shown in Table 1. Note that the ferromagnetic interaction is rather short ranged, and the effective exchange coupling parameter *J*_eff_ in 12 × 6 supercell is much smaller than that in 8 × 4 supercell. Moreover, the As or P impurities have a tendency to cluster which poses an upper limit to the useable concentration. Ferromagnetism at finite temperatures can thus only be expected at an appropriate As or P concentration.

## Conclusion

In summary, we have performed first-principles calculations to show that hole doping can induce ferromagnetism in SrS and SrSe monolayer sheets. From the electronic band structure calculations, the pristine SrS and SrSe monolayers are semiconductor with a flat band in the top valence band. As holes are injected into the SrS and SrSe monolayers, the system becomes ferromagnetic. With different hole densities, there are many possible phases including nonmagnetic semiconductor, half metal, magnetic semiconductor, and nonmagnetic metal. We also consider the p-type dopants in monolayer SrS and SrSe, i.e., substituting a single S (or Se) atom by an P (or As) atom. The magnetic moment, magnetic coupling strength and Curie temperature are calculated. A local moment of 1.0 μ_B_ is formed around the dopant atom, and the magnetic coupling between impurity-induced local moments is ferromagnetic. Our theoretical results provide valuable guidance for experimentalists to confirm and quantify the charge and spin phenomena in the SrS an SrSe monolayer materials. If synthesized, these novel 2D II-VI semiconductors may be useful for FET-based electronics, optoelectronics, and spintronics.

## Additional Information

**How to cite this article:** Lin, H.-F. *et al*. Magnetism in the p-type Monolayer II-VI semiconductors SrS and SrSe. *Sci. Rep.* **7**, 45869; doi: 10.1038/srep45869 (2017).

**Publisher's note:** Springer Nature remains neutral with regard to jurisdictional claims in published maps and institutional affiliations.

## Figures and Tables

**Table 1:** Magnetic properties of SrX (X = S, Se) monolayers substituted with P and As atoms.

System	Supercell	Ground state	Δ*E* (meV)	Jeff (meV)	*TC*^MFA^ (K)	*T*_C_ (K)
SrS	8 × 4	FM	98	49	187	114
	12 × 6	FM	81	41	154	94
SrSe	8 × 4	FM	154	77	286	175
	12 × 6	FM	136	68	252	154

The energy differences Δ*E*_FM−AFM_ between the ferromagnetic ground state and the antiferromagnetic metastable state. The effective exchange coupling parameter *J*_eff_. mean field Curie temperature *T*_C_^MFA^ of the ferromagnetic state are estimated from our DFT total energy calculations. The Curie temperature *T*_C_ are estimate by using an empirical relation *T*_C_/*T*_C_^MFA^ = 0.61.
